# Successful Treatment of Xanthogranulomatous Cholecystitis Following Total Knee Replacement

**DOI:** 10.7759/cureus.44474

**Published:** 2023-08-31

**Authors:** Matthew League, John Eick

**Affiliations:** 1 Internal Medicine, Lincoln Memorial University-DeBusk College of Osteopathic Medicine, Harrogate, USA; 2 Internal Medicine, Methodist University Hospital, Memphis, USA

**Keywords:** postoperative cholecystitis, gallbladder histopathology, cholecystostomy, acute acalculous cholecystitis, xanthogranulomatous cholecystitis

## Abstract

Xanthogranulomatous cholecystitis (XGC) is a rare form of chronic gallbladder inflammation that most commonly presents as acute cholecystitis and is often mistaken for carcinoma of the gallbladder. This case details the hospital course and follow-up of a 77-year-old male who developed suspected acute acalculous cholecystitis (AAC) resulting in severe sepsis after elective left total knee arthroplasty (TKA). Histopathological findings after elective cholecystectomy later revealed XGC as the underlying etiology.

## Introduction

While xanthogranulomatous cholecystitis (XGC) is generally considered to be a rare form of chronic cholecystitis, patients primarily present with signs and features of acute cholecystitis. There are many factors that can cause cholestasis leading to XGC; however, the majority of cases are a result of gallstone obstruction of the cystic duct. Cholestasis causes increased intraluminal pressures within the gallbladder which can lead to ischemia, bacterial proliferation, sepsis, and shock [1]. XGC is characterized by the inflammatory destruction of Rokitansky-Aschoff sinuses (RAS) and invaginations in the gallbladder muscularis formed by increased intraluminal pressures. RAS are present in 90% of cholecystitis gallbladder specimens; however, in XGC, these sinuses rupture, releasing biliary and cholesterol pigments inside the gallbladder wall and forming xanthogranulomas via a florid granulomatous histiocytic inflammatory reaction [2]. XGC is identified by the presence of such xanthogranulomas which appear as yellow structures within the gallbladder wall.

XGC typically occurs in patients over the age of 50 and has a prevalence ranging from 1.3% to 1.9% in Europe and the Americas and 8.8% in India [2,3]. XGC most commonly presents as acute cholecystitis with right upper quadrant pain, an elevated white blood cell count, nausea, vomiting, and fever [4]. Given its similarities in presentation to gallbladder carcinoma (GBC), proper diagnosis of XGC can be helpful in preoperative and intraoperative planning considering XGC has a known high risk of conversion to open cholecystectomy. While ultrasound and CT are typically first-line imaging, histopathological analysis should be performed via ultrasound fine-needle aspiration cytology for a definitive diagnosis in cases suspicious of GBC [5,6].

Since XGC is typically a histopathological diagnosis, it is often only found incidentally upon postoperative histopathological analysis after cholecystectomy, as we discovered in our case. This report aims to bring awareness to an uncommon acalculous presentation of XGC occurring after orthopedic surgery, as well as to highlight the successful management of an elderly patient with sepsis secondary to suspected acute acalculous cholecystitis (AAC).

## Case presentation

A 77-year-old Caucasian male with a past medical history significant for hypertension, gastroesophageal reflux disease, chronic gout, and mild neurocognitive impairment presented to our hospital’s emergency department from an inpatient rehabilitation facility with complaints of right upper quadrant abdominal pain, worsening lethargy, and left knee pain one week after elective left total knee arthroplasty (TKA). He underwent TKA due to progressive osteoarthritis but was otherwise in good health and able to complete all activities of daily living without significant issues. The rehabilitation facility from which he was being sent had started empiric vancomycin and cefepime due to concern for sepsis. The patient described the abdominal pain as sharp and feeling like a pulled muscle in his right upper quadrant.

The abdominal pain was associated with hypotension (92/63 mmHg), tachycardia (105 bpm), a respiratory rate of 18 respirations/min, and an oxygen saturation of 100% on two liters of oxygen via nasal cannula. On exam, the patient presented with an ill appearance, labored respirations, and obtundation, responsive only to noxious stimuli. He had right upper quadrant abdominal tenderness to palpation. His left knee incisions were clean, dry, and without any signs of infection. Initial laboratory evaluation was notable for leukocytosis, with a WBC count of 26.5 thou/mcl. Blood cultures obtained at admission returned positive results for coagulase-negative staphylococcus, which was suggested by pathology to likely be due to contamination. Additional lab values are shown below in Table 1.

**Table 1:** Laboratory values at hospital admission WBC: white blood cell, AST: aspartate transaminase, ALT: alanine transaminase, H: high, L: low

Test	Result	Reference range
Sodium (mmol/L)	131 L	135-145
Potassium (mmol/L)	4.1	3.5-5.0
Glucose, serum (mg/dL)	126 H	70-100
Creatinine (mg/dL)	1.26	0.6-1.3
Total bilirubin (µmol/L)	0.9	0-24
Alkaline phosphatase (IU/L)	88	44-147
AST (U/L)	52 H	8-33
ALT (U/L)	24	4-36
WBC (thou/mcL)	26.5 H	4-11
Hemoglobin (g/dL)	10.3 L	13-17
Hematocrit (%)	29.8 L	41-50
Platelet count (x10^9^)	298	130-400

**Figure 1:**
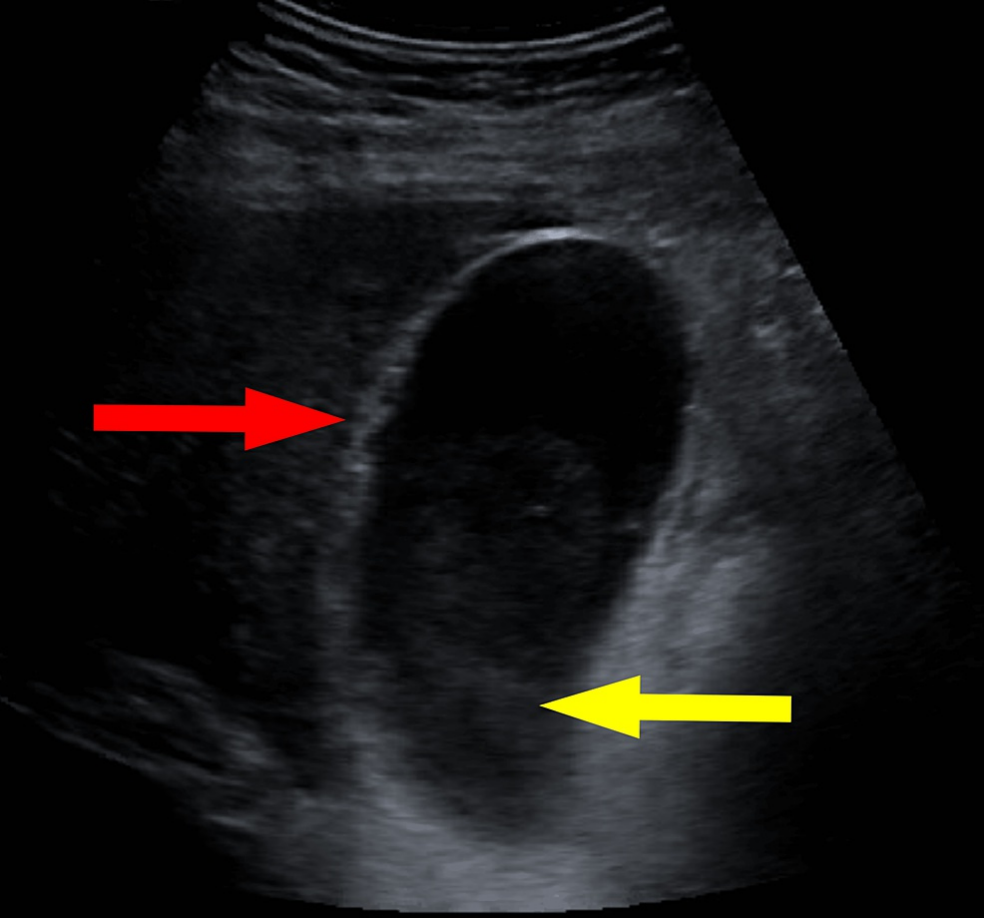
Ultrasound of the gallbladder Ultrasound of the gallbladder demonstrating sludge in the gallbladder (yellow arrow), mild wall thickening (red arrow), and absence of echogenic stone. These findings are suggestive of AAC

**Figure 2:**
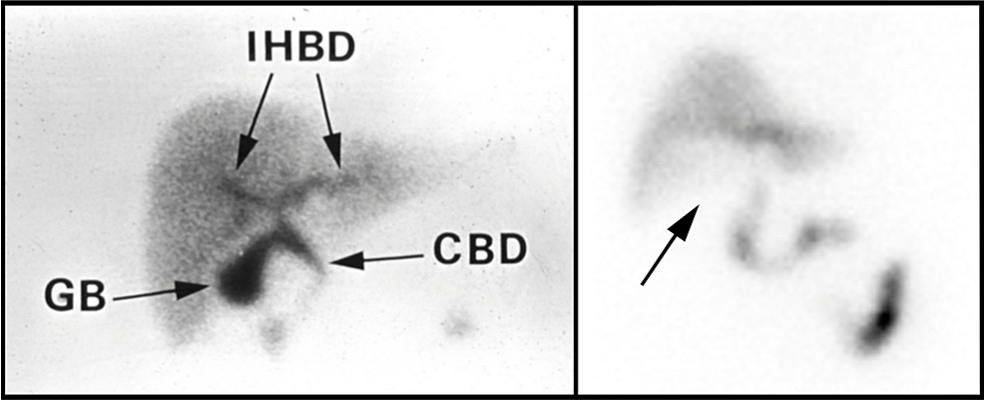
Nuclear medicine HIDA scan A normal HIDA scan image (left) showing distinct uptake of radioactive tracer throughout the biliary system [7]. This is contrasted with our patient’s abnormal HIDA scan (right) demonstrating nonvisualization of the gallbladder (black arrow) due to obstruction of the gallbladder outlet from biliary sludge. IHBD: intrahepatic bile ducts, GB: gallbladder, CBD: common bile duct

Abdominal ultrasound showed sludge in the gallbladder without echogenic stone and mild wall thickening, suggesting the possibility of AAC (Figure 1). A hepatobiliary iminodiacetic acid (HIDA) scan was then performed which showed nonvisualization of the gallbladder, a finding also compatible with a diagnosis of AAC (Figure 2). Since the patient was deemed to be a poor surgical candidate for cholecystectomy due to severe sepsis, a decision was made to place a cholecystostomy tube and continue antibiotic therapy. Interventional radiology then placed a 10-French pigtail drain percutaneously under CT guidance into the gallbladder. Over the following days, the patient’s condition improved. He was cleared for discharge on hospital day eight on a continued antibiotic regimen of amoxicillin-clavulanate 500-125 mg. At that time, the patient was transported to an inpatient rehabilitation center to continue postoperative physical therapy for his knee replacement.

Approximately five months later, the patient followed up with outpatient general surgery due to continued right upper quadrant abdominal pain and elected to undergo cholecystectomy. During laparoscopy, a considerable amount of adhesions were noted, especially the omentum which was adhered to the liver, and the hepatic flexure of the colon which was scarred to the gallbladder. Once the gallbladder was resected, the cholecystostomy tube was found to be within a peri-gallbladder hepatic abscess. Upon gross inspection, the gallbladder was markedly fibrotic and contracted in appearance. A subtotal cholecystectomy approach was used, and the gallbladder was successfully removed laparoscopically. Surgical pathology reported no evidence of gallstones. In addition, fungal stains for fungal forms (GMS, PAS-LG) and calcium (von Kossa) were all negative. After completed pathological analysis, a final diagnosis of XGC was given.

## Discussion

Our patient presented with sepsis one week following an elective left total knee replacement. Ultrasound and HIDA scan imaging suggested AAC as the source of infection, which was treated with antibiotics and drainage via a percutaneous cholecystostomy tube. Eventually, the patient elected to undergo elective cholecystectomy, which showed no evidence of gallstones. Pathological analysis revealed the presence of a hepatic abscess and XGC.

Our case represents a minority presentation of XGC given the lack of gallstone obstruction. One study showed that cholelithiasis was found in 69% to 96% of cases and obstruction of the cystic duct in 80% of cases [8]. On the contrary, the most common intraoperative features of XGC are thickening of the gallbladder wall (82% of cases) and adhesions to the surrounding organs (79% of cases) which we did find in our case [9]. Furthermore, the fibrosis and contraction of the gallbladder noted during surgery are consistent findings with the expected course of chronic cholecystitis over time.

Given the histopathological confirmation of XGC, we suspect that the knee replacement surgery was likely an exacerbating factor in the gallbladder inflammatory disease process. Since there are no clearly documented risk factors for XGC, we looked at risk factors for AAC. Risk factors for AAC are processes that contribute to cholestasis and increased intraluminal pressures, mechanisms that are also thought to contribute to XGC [2,10]. Thus, we theorize that undergoing non-biliary surgery, mechanical ventilation, and trauma pertaining to the routine knee replacement procedure may have contributed to the onset of XGC [11]. Further studies targeting the identification of XGC risk factors would be necessary to support this theory.

We were unable to identify any other documented cases of XGC occurring in a postoperative setting. Similar forms of cholecystitis, such as AAC, have been known to occur postoperatively, and a small number of studies document AAC following orthopedic procedures. One case report had a confirmed histological analysis of acute acalculous gangrenous cholecystitis, and the other reported acute or chronic acalculous cholecystitis with associated hemorrhage on pathological analysis [12,13]. Further study of postoperative acute cholecystitis would need to be done to better understand the epidemiology of XGC presenting postoperatively.

Cholecystectomy remains the recommended treatment of XGC, though there is a high risk of conversion to open laparotomy and other surgical complications. Biliary fistulas are the most common postoperative complication followed by surgical site infections, hepatic abscesses, bleeding, and organ injury. In the management of XGC complications, a partial liver resection may be necessary if local invasion has occurred due to fistula or abscess formation [5].

## Conclusions

XGC proves to be a difficult preoperative diagnostic challenge given its similar presentation to other forms of acute and chronic cholecystitis and the necessity for histopathological analysis to confirm its diagnosis. Though XGC is generally considered benign, it can result in cholecystitis leading to life-threatening sepsis. In its acute acalculous presentation, XGC should be treated as AAC until proven otherwise with surgical consultation for cholecystectomy versus percutaneous cholecystostomy and antibiotics. Cholecystectomies involving XGC should be approached with extreme caution and careful planning as they are at high risk of conversion to open due to the inflammatory nature of XGC and its propensity to form fistulae with surrounding organs.
